# Anterior cervical discectomy and fusion versus hybrid surgery in multilevel cervical spondylotic myelopathy: A meta-analysis: Erratum

**DOI:** 10.1097/MD.0000000000012618

**Published:** 2018-09-21

In the article, “Anterior cervical discectomy and fusion versus hybrid surgery in multilevel cervical spondylotic myelopathy: A meta-analysis”,^[1]^ which appeared in Volume 97, Issue 34 of *Medicine*, Dr. Wen-Yuan Ding should not appear as a corresponding author. The only corresponding author is Dr. Chun-Ming Zhao.
